# Optimal intervention time and risk of the activating blood and removing stasis method in acute cerebral hemorrhage patients: A randomized placebo-controlled trial

**DOI:** 10.1097/MD.0000000000024214

**Published:** 2021-01-15

**Authors:** Ying Ma, Dongmei Zhang, Zhiguo Lv, Yabin Cui, Yutong Fei, Tianying Chang, Mingkun Yu, Jing Lu, Qingxia Huang, Ying Zhang, Peng Xu, Tianye Lan, Jian Wang

**Affiliations:** a College of Traditional Chinese Medicine, Changchun University of Chinese Medicine; b Scientific Research Office; c Department of Encephalopathy, The Affiliated Hospital to Changchun University of Chinese Medicine, Changchun; d Centre for Evidence-Based Chinese Medicine, Beijing University of Chinese Medicine, Beijing; e GCP Department; f Research Centre of Traditional Chinese Medicine, The Affiliated Hospital to Changchun University of Chinese Medicine, Changchun, China.

**Keywords:** activating blood and removing stasis, cerebral hemorrhage, protocol, stroke

## Abstract

**Introduction:**

Stroke is the leading cause of disability-adjusted life years in neurological diseases and has become one of the top 3 fatal diseases in the world. Cerebral hemorrhage accounts for approximately 18% to 24% of all strokes in Asian countries. Cerebral hemorrhage is one of the most destructive subtypes of stroke and has high morbidity and mortality. Based on the current research, it has been confirmed that neither surgical treatment nor current drug treatment is the most preferred treatment. Traditional Chinese medicine (TCM) is increasingly being used to treat cerebral hemorrhage, and the activating blood and removing stasis (ABRS) method has received more attention. At present, there is still a lack of high-quality clinical research on the treatment of acute cerebral hemorrhage.

**Method:**

We designed a multicenter, prospective, randomized, double-blind, placebo-controlled clinical trial. We aim to recruit 312 cerebral hemorrhage patients aged 18 to 80 years within 24 to 72 hours after onset. In addition to routine treatment, participants will randomly receive ABRS granules or placebo for 14 days. Those enrolled within 24 to 48 hours after onset will enter strata A, and those enrolled within 49 to 72 hours (including 48–49 hours) after onset will enter strata B. The strata sample size ratio will be 1:1. The primary outcome is the disability degree (modified Rankin Scale score, mRS) at 6 months after onset. The secondary outcomes include the percentage of hematoma enlargement after treatment, Barthel index (BI), National Institutes of Health stroke scale (NIHSS) score, mortality rate, all-cause mortality rate, TCM stroke syndrome evaluation scale score, and adverse events.

**Discussion:**

The study is expected to confirm the safety and effect of acute cerebral hemorrhage within 24 to 72 hours treated with the ABRS method and to determine the optimal time for intervention in this period.

**Trial registration number:**

ChiCTR1900022627

## 1 Introduction

Stroke is the leading cause of disability-adjusted life years in neurological diseases and has become one of the top 3 fatal diseases in the world.^[1]^ Cerebral hemorrhage accounts for approximately 18% to 24% of all strokes in Asian countries, which is higher than that in Western countries.^[2]^ Cerebral hemorrhage is one of the most destructive subtypes of stroke and has high morbidity and mortality.^[3]^ The median mortality rate within 1 month was 40.4%, and the functional dependence after cerebral hemorrhage was approximately 75%.^[4]^ At the same time, it was found that hematoma enlargement was closely related to prognosis.^[5,6]^ Approximately one-third of patients with cerebral hemorrhage have hematoma growth within 24 hours.^[7,8]^ However, there is no specific treatment to prevent hematoma enlargement and promote hematoma absorption to improve the prognosis. In addition, based on the current research, it has been confirmed that neither surgical treatment nor current drug treatment is the most preferred treatment.^[9,10]^

In the face of the limitations of the treatment for cerebral hemorrhage, traditional Chinese medicine (TCM) is increasingly used to treat cerebral hemorrhage.^[11,12]^ According to the theory of traditional Chinese medicine, “abnormal flow of the blood is stasis,” so stasis occurs throughout the pathological process of cerebral hemorrhage. Therefore, the key to the treatment of cerebral hemorrhage is activating blood and removing stasis. At present, there are studies focusing on the therapeutic effect of activating blood and removing stasis, but there is still a lack of high-quality clinical research on the treatment of acute cerebral hemorrhage.

We designed a multicenter, prospective, randomized, double-blind, parallel controlled clinical trial to evaluate the optimal intervention time, efficacy and safety of the activating blood, and removing stasis method for acute cerebral hemorrhage within 24 to 72 hours after onset.

## 2 Methods and design

### 2.1 Study design and setting

**Figure 1:**
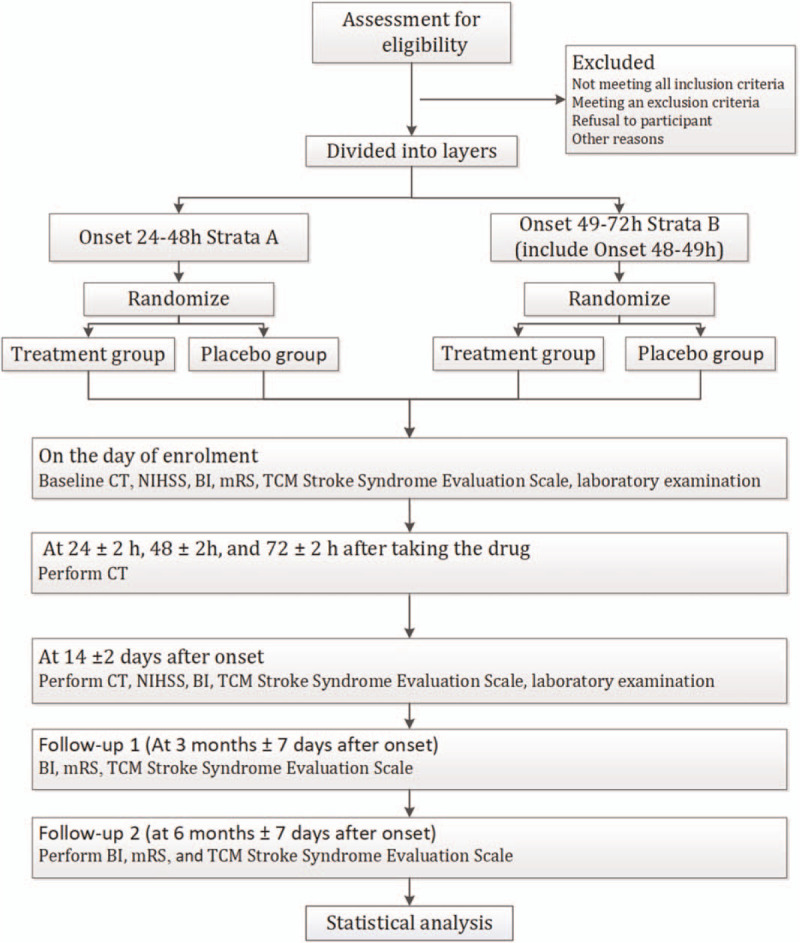
Study design diagram. BI = Barthel index, mRS = modified Rankin scale, NIHSS = National Institutes of Health stroke scale, TCM = traditional Chinese medicine, TCM = traditional Chinese medicine.

The study is a multicenter, prospective, randomized, double-blind, placebo-controlled clinical trial. Participants will be randomly assigned to the activating blood and removing stasis (ABRS) granule group or placebo group. Group allocation ratio is 1:1. The patients were stratified according to the time of enrollment. Patients who enrol within 24 to 48 hours after onset will enter strata A, and patients who enrol within 49 to 72 hours (including 48–49 hours) after onset will enter strata B. The strata sample size ratio is 1:1. Participants will receive ABRS granules in addition to routine treatment or placebo in addition to routine treatment. The treatment cycle is 14 days. The primary outcome is disability degree (modified Rankin scale score). This study is reported in accordance with the Standard Protocol Items: Recommendations for Interventional Trial (SPIRIT) guidelines.^[13]^ The study flow chart is shown in Fig. 1.

We will conduct this study in 7 hospitals in China. These hospitals are The First Hospital of Jilin University, The Second Hospital of Jilin University, China-Japan Union Hospital of Jilin University, Dongfang Hospital Beijing University of Chinese Medicine, Jilin Province People's Hospital, Jilin Hospital of Integrated Traditional Chinese and Western Medicine in Jilin Province, and Liaoyuan Traditional Chinese Medicine Hospital.

### 2.2 Study objectives

The objective is to investigate the efficacy and safety of the ABRS method for acute cerebral hemorrhage and to determine the optimal intervention time.

### 2.3 Eligibility criteria

#### 2.3.1 Inclusion criteria

1. Diagnosis with cerebral hemorrhagic stroke confirmed by computed tomography (CT).
2. Age between 35 and 80 years old.
3. Stroke onset time within 24 to 72 hours.
4. Receive conservative medical treatment.
5. Glasgow coma score >6 points.
6. mRS score before onset of 0 to 1 point.
7. Informed consented.

#### 2.3.2 Exclusion criteria

1. Cerebral hemorrhage was confirmed to be caused by a brain tumor, blood disease, or brain trauma (or postoperative).
2. Coma or cerebral hernia at the onset of cerebral hemorrhage.
3. Inability to take the study drug orally or nasally because of gastrointestinal bleeding.
4. Emergency surgery.
5. Subarachnoid hemorrhage.
6. Pregnancy or lactation.
7. Severe primary disease, such as cardiovascular, hepatic, renal and hematopoietic disease, mental disease, or abnormal (double the normal level) results on routine coagulation, liver function, and kidney function evaluations.
8. Allergic constitution or known allergy to alcohol or ingredients of TCM ABRS granules.
9. Hemorrhagic stroke induced by anticoagulant drugs.
10. Participation in other clinical trials in the last 3 months.

### 2.4 Recruitment

Inpatients with cerebral hemorrhage in all recruiting centers will undergo screening assessments to determine their eligibility. Patients meeting all of the inclusion criteria and none of the exclusion criteria will be informed of this study. All participants will be fully informed of the purpose, process, other treatment options, risk and benefit of the study, and sign an informed consent form if they agree to participate.

### 2.5 Randomization and treatment allocation

Randomization will be performed immediately after signed informed consent has been obtained. This study will use a stratified block randomization design, with stratification by recruiting center and time of enrollment. Randomization will be performed by the Center for Evidence-Based Chinese Medicine of Beijing University of Chinese Medicine using SAS version 9.2 (Cary, NC). Participants will be randomized at a 1:1 ratio to the ABRS granule group or the placebo group.

### 2.6 Blinding

The study will be a double-blinded clinical trial. All personnel, researchers, care providers, and participants will remain blinded to the intervention being received. The placebo is identical in appearance, smell, and packaging to the ABRS granules. The indications for deblinding are serious adverse events (SAEs) as instructed by the local medical ethical review committee or other dire emergencies. We set up emergency code-breaking envelopes for deblinding for individual participants.

### 2.7 Intervention

In addition to routine treatment (including general treatment, control of blood pressure, reduced intracranial pressure, and so on) according to the guidelines, participants will immediately receive ABRS granules or placebo after randomization. The preferred method of intervention will be oral; if the patient cannot swallow normally, the drug will be given nasally. The dosage will be 1 packet (15 g) twice a day for 14 days. The ABRS granule or placebo will be completely dissolved in 150 mL of boiling water before administration. The ABRS granule contains 8 kinds of herbs. The placebo was made from 5% ABRS granule and dextrin. The ABRS granules and placebo were produced and packed by China Resources Sanjiu Medical and Pharmaceutical Co., Ltd., Shenzhen, China, and passed the quality inspection before leaving the factory. The researchers have completed unified training and will patiently communicate with participants to increase compliance. Participants with <80% medication compliance will be excluded.

### 2.8 Concomitant medications

After randomization, other traditional Chinese medicines and treatment methods for treating stroke or having ABRS function should be avoided during treatment. The name, usage, and dosage of medicines that must be continued for other diseases will be recorded in the case reporting form (CRF).

### 2.9 Outcome measures

#### 2.9.1 Primary outcome

The primary outcome will be the disability degree (mRS score) at 6 months after onset. The mRS is widely used to assess disability in stroke patients.^[14]^ The mRS divides the disability degree into 6 grades, and the score ranges from 0 to 5. A higher mRS score indicates a more serious degree of disability.

#### 2.9.2 Secondary outcomes

The secondary outcomes included the percentage of hematoma enlargement after taking the drug. After taking the medicine, participants will undergo 4 follow-up CT scans to collect information about the effect of the ABRS method on hematoma absorption or enlargement. Other secondary outcomes include the Barthel index (BI), National Institutes of Health stroke scale (NIHSS) score, mortality rate, all-cause mortality rate, TCM stroke syndrome evaluation scale, and adverse events.

### 2.10 Safety assessment

Safety will be evaluated according to the incidence of adverse events and the results of clinical laboratory examinations. All adverse events will be recorded. The possible association between adverse events and the intervention will be determined according to a 5-level classification: affirmative, probable, possible, suspicious, and impossible.

Any adverse events during the trial will be recorded using the “adverse event form” and continue to be investigated. Researchers will record the treatment process if needed and results until the laboratory examination results return to normal and symptoms and signs disappear. All SAEs will be reported to the research ethics committee within 24 hours. When participants become unsuitable to continue the trial due to adverse events, they will be withdrawn from the trial immediately.

Clinical laboratory examinations will include routine blood, urine, stool + occult blood, liver function, kidney function, coagulation function, glucose and D-dimer tests, and electrocardiography.

### 2.11 Data collection and management

The trial uses the CRF to collect on the day of enrollment, at 24 ± 2 hours, 48 ± 2 hours, and 72 ± 2 hours after taking the medicine, 14 ± 2 days after onset. The 2 follow-up visits were carried out at 3 months ± 7 days and 6 months ± 7 days after onset. More information about the study visits is shown in Table 1.

**Table 1:** Study schedule.

		After taking the medicine				Follow-up	
Assessments	Enrollment	24 h	48 h	72 h	14 days after onset	3 months after onset	6 months after onset
Informed consent	×						
Randomization	×						
Medical history	×						
Physical examination	×				×		
General demography	×						
CT scan	×	×	×	×	×		
TCM stroke syndrome evaluation scale	×				×	×	×
NIHSS	×				×		
BI	×				×	×	×
mRS	×					×	×
Routine blood test	×				×		
Routine urine test	×				×		
Routine stool + occult blood test	×				×		
Liver and kidney function tests	×				×		
Coagulation function and D-dimer tests	×				×		
Glucose test	×				×		
Electrocardiography	×				×		
Distribution of drugs	×						
Recovery after drug use					×		
Recording of combined medications	×				×		
Recording of adverse events	×				×		
Safety assessment					×	×	
mortality					×	×	×

BI = Barthel index, mRS = modified Rankin scale, NIHSS = National Institutes of Health stroke scale, TCM = traditional Chinese medicine.

The participant's name, medical records, and data collected relating to the study will remain confidential information. Participants will be identified for the purposes of the study by initials and an assigned participant number. To ensure the security of the data, study-related information will be stored in a locked file cabinet in a safe place that cannot be accessed without permission.

To ensure the authenticity, accuracy, and completeness of the data, the quality of the data will be reviewed on site by a monitor through 100% source data verification. Errors will be submitted to the researchers for correction. After the observation course of the participant, the researcher submitted the CRF, informed consent, and medication record of the participant to the main researchers of their recruiting centers for review within 3 working days and submitted these study files to the project leader for review within 1 week.

### 2.12 Sample size

The sample size was calculated by PASS software version 15 (Kaysville, UT). In a previous study, the rate of unfavorable disability (according to the mRS score) in the control group was 56%.^[15]^ We assume that the rate of unfavorable disability (according to the mRS score) in the treatment group will be 36%, that is, decreased by 20%. After a 2-sided Z test, PASS software calculated that the sample size of each group should be 129. Considering a 20% dropout rate, 312 patients will need to be enrolled.

### 2.13 Statistical analysis

Inferential statistical analyses as specified will be conducted, and all comparisons will be between the ABRS granule group and the placebo group. The statistical analyst will be blinded to the participants’ personal information and group assignment during the trial. The primary analysis will be based on the full dataset following the intention-to-treat analysis principle for all efficacy outcomes, while the secondary analysis will be based on the per protocol dataset. For the analysis of safety outcomes, a safety dataset will be used. The primary outcome will be determined by a comparison of the changes in the patients’ mRS scores between the 2 groups at 6 months after onset. The secondary outcomes will include the mortality rate, hematoma enlargement percentage, all-cause mortality rate, adverse events, TCM stroke syndrome evaluation scale score, BI, and NIHSS score. The hematoma enlargement percentage will be measured at baseline, 24, 48, and 72 hours after taking the medicine and 14 days after onset. The mortality rate, all-cause mortality rate, BI, and TCM stroke syndrome evaluation scale score will be measured at 14 days, 3 months, and 6 months after onset. The NIHSS score will be measured at 14 days after onset. The mRS score will be measured at 3 and 6 months after onset.

Missing data regarding the primary and secondary outcomes will be imputed using the last observation carried forward (LOCF) method. Independent *t* tests or Wilcoxon rank-sum tests will be used to analyze continuous outcomes. Chi-squared tests will be used to analyze categorical data. Analysis of variance or a generalized linear mixed model will be used for repeated measurement data. Regarding mortality, survival probabilities will be estimated using the Kaplan–Meier method. Two-sided 95% confidence intervals will be constructed for assessment, and a 2-sided alpha level of 0.05 will be used to indicate significance. Adjustments will be made for baseline imbalances. Descriptive statistics, including the number and percentage for categorical variables and the number, mean, standard deviation, median, minimum and maximum for continuous variables, will be provided. Listings of individual patient data will also be produced. A comprehensive statistical plan will be prepared and finalized prior to unblinding and analysis of the data.

## 3 Discussion

Cerebral hemorrhage usually has greatly negative effects and places a large economic burden on patients and their families. In recent years, under the guidance of TCM theory, ABRS has been widely used in the treatment of cerebral hemorrhage. Several animal experiments have verified the benefits of TCM for cerebral hemorrhage. Experiments have found that TCM herbs decrease oxygen and glucose deprivation-induced Ca2+ overload and endoplasmic reticulum stress while inhibiting apoptosis by decreasing mitochondrial damage.^[16]^

Previous studies have provided some evidence about the efficacy and safety of treating cerebral hemorrhage with TCM, but most of them have been literature reviews.^[17,18]^ Some clinical trials have verified the safety of TCM in acute cerebral hemorrhage, but most of these have been retrospective studies or randomized controlled studies with a small sample.^[19,20]^ Thus, a high-quality trial of the ABRS method in acute cerebral hemorrhage is necessary.

Previous randomized controlled trials have often focused on the efficacy and safety of the ABRS method for acute cerebral hemorrhage while neglecting to explore the optimal timing of the intervention. The overall trend shows that patients with acute cerebral hemorrhage can gain more benefits from adjuvant treatment with the ABRS method than from treatment with conventional Western medicine alone. However, previous randomized controlled trials of the ABRS method applied within 6 hours showed that TCM treatment was not beneficial and increased the risk of hematoma enlargement.^[21]^ At the time of writing, there are no multicenter, large-sample, prospective, double-blind, placebo-controlled randomized controlled trials exploring the best timing of intervention with the ABRS method for acute cerebral hemorrhage. This study will fill in the gap in this area and facilitate clinical decision-making.

However, this trial will also have some limitations. To obtain a more representative patient population, we will recruit participants from 7 centers. However, the study will be carried out only in China. Therefore, whether the test results can be applied in all countries needs to be further verified.

The results of this study will be of importance for patients with cerebral hemorrhage, as they will allow us to draw conclusions on the efficacy, safety, and optimal timing of intervention with the ABRS method. This study may provide clinical evidence as a basis for the treatment of acute cerebral hemorrhage with the ABRS method and improve the prognosis and quality of life of patients with cerebral hemorrhage.

## Acknowledgments

The authors appreciate the support and efforts of those who have been or will be included in this study.

## Author contributions

**Conceptualization:** Tianye Lan, Jian Wang.

**Formal analysis:** Yutong Fei, Mingkun Yu.

**Funding acquisition:** Jian Wang.

**Investigation:** Jing Lu, Qingxia Huang, Ying Zhang.

**Project administration:** Dongmei Zhang, Jian Wang.

**Supervision:** Yabin Cui, Peng Xu, Tianye Lan.

**Visualization:** Ying Ma, Dongmei Zhang.

**Writing – original draft:** Ying Ma.

**Writing – review & editing:** Zhiguo Lv, Tianying Chang.

## Abbreviations

Abbreviations: ABRS = activating blood and removing stasis, BI = Barthel index, CRF = case reporting form, CT = computed tomography, mRS = modified Rankin scale, NIHSS = National Institutes of Health stroke scale, TCM = traditional Chinese medicine.
